# MD-MAC: A Distributed TDMA Protocol Based on Desynchronization for Multi-Hop Topologies

**DOI:** 10.3390/s19235102

**Published:** 2019-11-21

**Authors:** Chaoyi Zheng, Shengchun Huang, Jibo Wei, Qiangjian Dong

**Affiliations:** School of Electronic Science, National University of Defense Technology, Changsha 410073, China; huangsc@nudt.edu.cn (S.H.); wjbhw@nudt.edu.cn (J.W.); dongqiangjian@nudt.edu.cn (Q.D.)

**Keywords:** Ad Hoc network, TDMA scheduling, time slot allocation

## Abstract

Communication resource allocation and collision detection are important for the Ad Hoc network. Considering the existing TDMA-MAC protocol, the allocation way based on fixed time slot is mostly adapted, which cannot guarantee the performance and be not flexible about the business for different nodes in the distributed network. Desynchronization, as a biological term, can be utilized in the Ad Hoc network. It implies that sensor nodes interleave periodic events to occur in succession through negotiation and adjustment. In this paper, we design a MAC protocol(MD-MAC) in the multi-hop network based on the idea of Desynchronization to solve the problem caused by stale information and lay down the adjustment rule to allocate the communication resource. Also, we propose a scheme which the network can detect collision in a self-adapting way. Finally, we simulate the proposed protocol to evaluate the performance. The experimental results indicate that the proposed algorithm can accelerate the convergence speed of resource allocation, solve collision and improve the efficiency of the distributed network.

## 1. Introduction

Since mobile Ad hoc network is used quite frequently, more and more things are connected into information space to intercommunicate and cooperate with each other [1,2]. Physical space is connected into information space by various sensing technologies [3]. The importance of Ad Hoc network promotes that some innovative applications have been proposed to improve network connectivity and data delivery ratio. For example, Unmanned Aerial Vehicles (UAVs) implementation receives increasingly attention. The Flying ad hoc networks (FANET) consists of plenty of UAVs, which communicates in a self-adapting way. Thus, the network connectivity is our concern part. The design of Media Access Control (MAC) protocol aims at optimizing the communication performance through formulating the strategies nodes use to access the common channel [4]. The ideal MAC allocation protocol can make the effective use of communication resources to improve network adaptability and prolong network lifetime [5]. Therefore, the resource allocation is of important for the UAVs network. In general, the base station is existed in the traditional communication framework, which connects with all users. However, for the UAVs swarm system, it limits the inflight range and cannot solve the disconnected problem. Therefore, the solution adopted is to establish the Ad Hoc network between different users, namely FANET. However, compared with traditional communication network, the drawbacks of the FANET are the limited communication resources, varying degrees of business demand and highly requirement for the network connectivity, which can not adopt traditional wireless communication protocol and need to be focused while designing the Mac algorithm. Generally, the MAC protocol can be divided into two categories: competition and allocation. Usually, the system of the FANET consists of distributed topologies, which means there is no central node in the network. Adopting a protocol based on competition will cause a plenty of collision and get a low bound throughput. Hence, the protocol based on allocation is a great choice. According to the previous study [6], the distributed TDMA protocol is appropriate to the demand of the FANET.

In the existing study, researchers proposed plenty of TDMA protocols which are about topology-transparent scheduling schemes and topology-based scheduling schemes. Rhee et al. [7] proposed the DRAND scheme which is a randomized distributed time slot scheduling algorithm. It allocated the time slot sequence by coordinating requests among network node. However, the complete randomness decreased the efficiency and resulted in the high collision rate of the message in the distributed process. Based on the DRAND, Li et al. [8] made improvement and proposed the E-T DRAND, which allocated time slot through the remainder energy and topology factor for each node, the results showed that there was a certain improvement in the scheduling rate and an appropriately decrease in the overhead. Bryan et al. [9] proposed a distributed time slot allocation protocol based on a new Graph Coloring technology called DSA-CCH. It can achieve a measure of parallelism in the case of not using global information to color. Liu et al. [10] focused on the interaction between the MPR physical layer and MAC layer and proposed a topology-transparent algorithm, i.e., m-MPR-l-code algorithm which can take full advantage of the MPR capability to improve the network performance. The result showed that the algorithm had a certain improvement on the performance of resource scheduling. However, these approaches introduce complicated polynomial-based algorithms and an important problem is that it exits redundant slots not to be utilized efficiently. The length of time slots allocated in the protocols mentioned above is fixed and the process is discrete, which causes the waste of communication resource. In the meanwhile, a lot of bionic protocols were proposed, including Frog call-inspired [3], simulated annealing algorithm [11], Particle Swarm Optimization algorithm [12] and so on. In 2007, Degesys et al. [13] observed firefly firing and proposed an one-hop TDMA algorithm based on DESYNC. The algorithm assumed that all nodes know global information so that each node can adjust according to its neighbors information and converge to a stable status. In this protocol, the slot length occupied by every node is suitable for its demand and the allocation process is continuous. In 2017, Kim [14] made improvement about the algorithm and proposed a firing offset adjustment scheme for increasing the efficiency of time slot utilization. However, the whole system was still bound by one-hop system. In 2018, Mühlberger et al. [15] presented the refractory threshold as an additional parameter to solve the stale information problem for multi-hop topologies, but it can be effective partly. Also, there must exist transmission collision while the topology changes. Z-MAC [16] assigns each node a slot in a TDMA manner. if one node has no data to transmit, its time slot will be occupied by other nodes with a contention way. However, due to the random choice and Inefficient backoff process, the protocol has a slow convergence speed. In 2016, Zhao [17] proposed E-Mac, which adjusts the structure of frame, backing off according to the collision part in the frame and solving the collision problem.

In this paper, we propose the MD-Mac algorithm which applies to the multi-hop network. The core idea of MD-Mac lies in that, every node transmits at least once in a given time cycle to adjust its own communication resource from local to global, and occupies the time slot larger than the minimum length. We modify the DESYNC mechanism to implement this idea and prove the effectiveness of the modification via both theoretical analysis and computer simulation. The main contributions are as follow:

1. The protocol we design is a totally distributed protocol, which does not rely on the prior knowledge of nodes(for instance, the busy time slots or the whole topology), and requires neither central node to negotiate or the global information.
2. Solving the problem of stale information, namely communication delay between each node, which makes the DESYNC process feasible in multi-hop networks.
3. A collision detection scheme is proposed, which can avoid collisions caused by topology change or multiple network mergence after the network converge.

The remainder parts of this paper are organized as follows: Section 2 introduces Desync-MAC protocol and the system model, then defining the problem of MD-MAC in the distributed network. Section 3 describes the process of the MD-MAC, and explains the principle, and then Section 4 explains the implement issues and the collision-free scheme. The performance of the proposed protocol is evaluated in Section 5 with computer simulations. Finally, we make conclusions in the last part.

## 2. System Model and Problem Definition

### 2.1. Desynchronization Process

**Figure 1:**
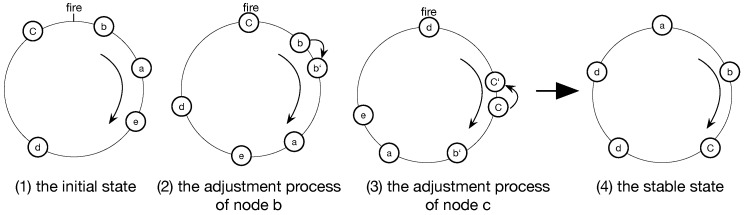
System model.

The DESYNC-based TDMA protocol is a centralized implementation which does not require a global clock and adjusts to the bandwidth of nodes in the network automatically. In the protocol, all nodes in the network can communicate with each other (i.e., they are in a fully-connected network). For understanding easily, every period can be considered as a ring and all nodes run in the ring cycle by cycle. The protocol assigns exclusive numbers for all nodes, so that nodes can be allocated to the period ring through their serial number (Figure 1). There is a firing event corresponding to a node broadcasting a firing message, which all nodes can hear, including its clock and time slot information. Therefore, nodes can send their firing message and listen to others in each period to adjust their states. In order to achieve this, node must pay attention to the message sent by neighbor nodes before and after its own. While the node stores firing messages from the previous and after nodes, it acquires message from its neighbors and adjusts its firing time to the phase midpoint of nodes before and after its firing time in the following period. For facilitating the understanding, the period usually can be seen as a circle and nodes are distributed in time sequence (as shown in Figure 1). When node *c* reaches the firing time(Figure 1(2)), it will send message to other nodes. node *b* starts to jump if information of the pervious and after nodes of node *b* in the ring (namely *a* and *c*) has been stored. Finally, after several cycles, each node moves its firing time to the midpoint of neighbors and the system will become stable (Figure 1(4)), namely desynchronized, which firing time of all nodes are distributed in the same interval.

The protocol, implementing in the single-hop network, has an advantage in the utilization of the bandwidth. Compared with other MAC protocols, Desync-MAC can provide excellent total throughput and collision-free station under high loads. Also, once the system keeps stable, regardless of the nodes leaving or adding, the system can be self-adapting to accommodate the new one or recapture the unoccupied time. However, Desync-MAC can not be suitable for all types of traffic since the communication demand of fully-connected network. For the WSNs, the distributed network without center node is normal, and nodes only connect with the one in their communication range. While there are many nodes distributing in the multi-hop network, a limitation is that each node should wait for firing messages sent by its neighbors which can not be sure that if connecting directly or through other nodes. Therefore, the process will be delayed and the stale information will impacts the packet transmission immediacy. In addition, the situation in which a crop of nodes leave or add in the multi-hop topology will cause the communication collision. In order to solve these problems in Desync-MAC protocol, we propose a distributed TDMA protocol which can be implemented in the multi-hop network, namely MD-MAC protocol which is the extension of Desync-MAC protocol. This protocol focuses on the accommodation of the universal traffic and solves the impact of stale information. Thus, nodes can negotiate with their neighbors to achieve the stable status in the multi-hop network.

### 2.2. MD-MAC System Model

**Figure 2:**
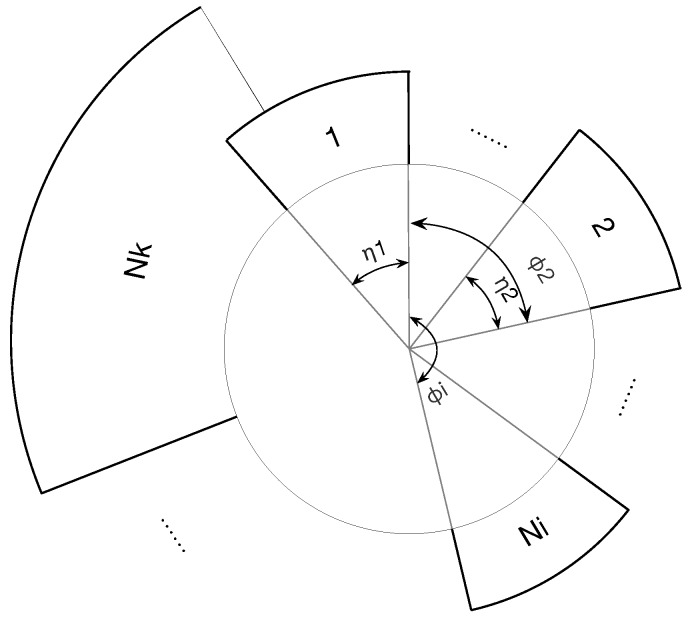
System model.

We consider a wireless communication network similar to the Ad Hoc network which contains *N* nodes, represented by $N_{i}$, i=1,2,…,n in the network. The topology of the network can be represented by an directed graph G(V,E), where V and E are the sets of nodes and edges respectively to indicate some nodes are connected. And we define that *D* and $D_{max}$ as the number and the maximum number of interferential neighbors. The rule is that each node can transmit at least once during a period, and does not support that packets send or receive in the meantime. Due to the interference range, every node can only transmit with its two-hop neighbors. Thus, nodes outside the interference range can occupy the same time slot in the meanwhile.As illustrated in Figure 2, each node occupies a period of time, namely length of the sector, and the ring consists of all nodes, whose length is less than a period. The system can be presented as Equation (3). All nodes acquire their exclusive serial numbers which are used to assist nodes to assign to the ring in clockwise order. We use ψ=($\phi_{i}$,$\eta_{i}$) to denote the state of node $N_{i}$, where $\phi_{i}\in \left[0,1\right]$ denotes the phase of $N_{i}$’s transmission, and $\eta_{i}$ denotes the transmission time it occupies. When $N_{i}$ reaches the firing time, i.e., $\phi_{i}$=1, $N_{i}$ will transmit the fire packet which informs $\psi_{i}$ to its neighbors, otherwise it will keep silence and its $\phi_{i}$ continues to increase. After the transmission, $N_{i}$ will reset its phase $\phi_{i}$ to 0. We should notice that if two nodes are not in communication range with each other (for example $N_{k}$ and $N_{1}$ in Figure 2), they can overleap their η with each other.

Different from the single-hop network, in this paper we assume that all nodes connect with their neighbors in a distributed way. The communication range we set is within two hops of nodes. When the transmission distance exceeds two hops, the information will be sent through nodes on routing. For the level-three (Network Layer) routing problem, the forwarded packet is the same as the one transmitted by a node in level-two (Mac Layer).

Also, the time synchronization problem can be solved by MTS algorithm [18] which is fully distributed, asynchronous and robust to dynamic network topologies. The clock model in the algorithm can be presented as:

(1) $$\begin{array}{c} \tau_{i}\left(t\right)=a_{i}t+b_{i} \end{array}$$

where $a_{i}$ is the clock skew and $b_{i}$ is the clock offset. In our protocol, the clock message of each node can be transmitted to its neighbors with its firing packet while reaching the firing point. According to the MTS algorithm, each node can calculate the relative skew and synchronize to a common clock iteratively. The basic idea of the MTS algorithm is to drive the logical clocks to the maximum value among all nodes to achieve a global synchronization, so it is suitable for our protocol to synchronize clock.

**Definition** **1.** *collision-free state.*

In the distributed network, the collision-free state means that no transmission for any two nodes in the interference range in the network overlaps. Formally, $N_{i}$ can experience no collision with the followed transmitter once Equation (2) is satisfied. Also, in the multi-hop network, the slots are allowed to reused by nodes with no interference, as shown in Equation (3).

(2) $$\Delta \phi_{i}=\phi_{i}-\phi_{i-1}\geq \eta_{i},\;i=1,2,\ldots ,n\;and\;\phi_{0}=\phi_{n}-1\geq \eta_{i}$$

(3) $$\sum_{i=1}^{n}\left(\phi_{i}-\phi_{i-1}\right)\geq 1,\;T\leq \sum_{i=1}^{n}\eta_{i}\leq D_{max}T$$

If all nodes satisfy Equation (2), it means that nodes in the network will transmit in clockwise order in the collision-free state. And the constraint is satisfied in the distributed network.

**Definition** **2.** *stale information.*

In the single-hop network, nodes receive information from other transmitters, so that the desynchronization process can be implemented and each node can select time slots with no error. However, the topology enlarges in the distributed network and nodes only know the information in the communication range, which cause transmission delay, namely stale information. The stale information can affect the network convergence distinctly. Hence, it is a big deal to alleviate the influence by stale information.

The problems which we focus on are that how nodes allocate time slots based on their own state and information in the distributed network, and deal with negative effects for stale information to make the length of slot to change continuously. When the topology changes, nodes can adjust their transmission point and slot length in a self-adapting way, and finally achieve collision-free state. Also, we would like to get a large enough throughput and a faster convergence speed.

## 3. Algorithm Description

In this section, we will describe MD-Mac in detail. Firstly, We set different serial number to all nodes, the same as the Desync-MAC protocol, for making nodes to select their own initial point randomly in clockwise order. The numbers for nodes are random and do not impact the distribution process. The initial slot length we set is η, and we can calculate the probability that all nodes do not overleap with their neighbors according to the following equation

(4) $$\begin{array}{c} p_{i}=\prod_{a=0}^{d_{i}}\left(T-a\eta \right)/T \end{array}$$

where $d_{i}$ is the number of degree of $N_{i}$, and *T* denotes the length of period. We make $p_{i}>95\%$ to calculate the limitation of η. But there is a deviation between the calculation result and the practical one, so another way is proposed.

For different topologies, the range of η can be set to $\eta \in (10^{-4},$T/($D_{max}$+1)) initially according to the length of the period *T* and the maximum number of degree $D_{max}$. Then we use the dichotomy way to make sure the limitation of η. If there are still collisions in the ring, we can use the E-Mac [17] scheme, which has been confirmed that is efficient to this condition, to solve the problem. However, in this case what should be noted is that the throughput of the network is low and the length of slots allocated to nodes will be changed continuously in the next step.

### The Process of MD-MAC

**Figure 3:**
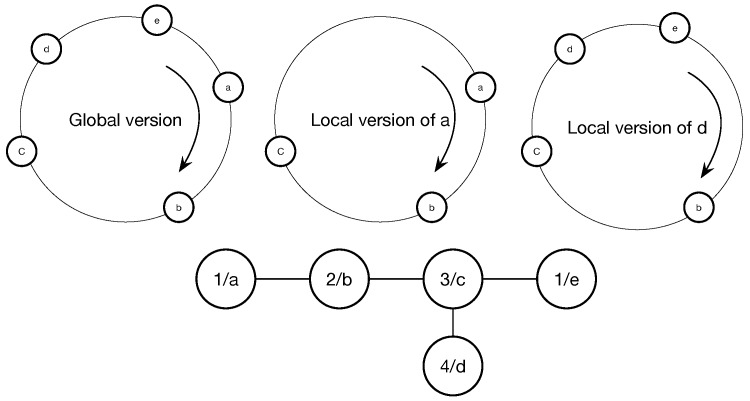
An example topology.

We adopt a simple idea to solve the aforementioned problem. The aim is that making nodes to negotiate with their neighbors and adjusting based on their own state and information to increase slot length which they occupy continuously. As shown in Figure 3, the ring of global view includes all nodes, but the ring of local view $N_{i}$ only includes the neighbors in its two-hop range, such as the local view of a and b in the figure.

Firstly, we rule that all nodes just send and receive packets in the first two periods and do not adjust their transmission time η and phase ϕ. In this way, every node can obtain information including its two-hop neighbors’ phases, the number of neighbors in the topology and in their local rings. From the third period, every node adds a time stamp in its frame which is set to 0 initially and transmits fire packet to its topology neighbors while its phase becomes to 1. When some node adjusts its phase, its time stamp $Ts_{i}\left(t\right)$ will plus one. All nodes should pay attention to the phases of their previous and next neighbor. Assuming that $N_{j}$ is $N_{i}$’s previous neighbor, and $N_{k}$ is $N_{i}$’s next neighbor, there are three conditions we distinguish for the multi-hop network:

1. $N_{j}$ and $N_{k}$ are one-hop neighbors of $N_{i}$:
  In this case, the same as the Desync-MAC protocol. Whatever the time stamp $Ts_{i}\left(t\right)$ is, $N_{i}$ adjusts its phase to the midpoint of $N_{j}$ and $N_{k}$ while ϕ of the after node is equal to 1, which means that $N_{i}$ has stored the current information of its neighbors.
2. Either of $N_{j}$ or $N_{k}$ is one-hop neighbor of $N_{i}$ in the topology, and another is two-hop:
  In this case, only if phase of $N_{i}$’s one-hop neighbor is to 1 and its time stamp $Ts_{i}\left(t\right)$ is equal to the two-hop node $Ts_{j}\left(t-1\right)$ or $Ts_{k}\left(t-1\right)$, $N_{i}$ starts to adjust.
3. Both of $N_{j}$ and $N_{k}$ are two-hop neighbor of $N_{i}$:
  In this case, the rule is that $N_{i}$ starts to adjust only when the phase of minimum node of $N_{i}$’s on-hop neighbor is set to 1 and both of neighbors’ time stamp $Ts_{j}\left(t-1\right)$ and $Ts_{k}\left(t-1\right)$ are equal to $Ts_{i}\left(t\right)$.

In the distributed network, delay is inevitable, so that keeping the neighbor information fresh is essential. According to the rule mentioned above, the node in case 1 will adjust firstly like the way in the DESYNC protocol and its time stamp will add one after the process. If the time stamp of the previous or next neighbor of a node $N_{i}$ is not less than $Ts_{i}\left(t\right)$, it is clearly shown that $N_{i}$ restores the immediate information of its neighbor, so $N_{i}$ just needs to judge the time stamp of another neighbor. Hence, the node in case 2 or 3 will move after the adjustment of its neighbors in case 1. After the adjustment for nodes in case 2 or 3, the node in case 1 will go on the next adjustment while the nodes in its local view have adjusted one time. Therefore, we can utilize the time stamp to make sure the real-time information restored by all nodes and prevent the network to divergency, the detail is shown as Algorithm 1.

**Algorithm 1** The Process of MD-MAC.
**Input:** node $N_{i}$, the previous neighbor $N_{j}$, the following neighbor $N_{k}$, time stamp *K***Output:** Initialization Each node initializes its time stamp       //add the time stamp **For** *t*=1 **to** *T* **While** t≤ 2       //the first two period, collecting neighbors information  Each node transmit with its neighbors and build its  neighbor table **endwhile** **If** t>2 **then**       //start to adjust  **For** *i*=1 **to** *N*   **If** $\phi_{i}=0$ **then**    $N_{i}$ send fire packet broadcast       //reach the firing point   **else**                                           //adjust phase according to different cases    **If** in case 1 & $\phi_{j}$ = 0 **then**     $N_{i}$ adjusts its phase to $\left(1-\alpha \right)\phi_{i}+\alpha \phi_{mid}$    **endIf**    **If** in case 2 & $\phi_{oh}=0$ & $K_{i}=K_{th}$ **then**     (oh means one-hop neighbor and tw means     two-hop neighbor)     $N_{i}$ adjusts its phase to $\left(1-\alpha \right)\phi_{i}+\alpha \phi_{mid}$    **endIf**    **If** in case 3 **&** $\phi_{nearest}=0$ **&** $K_{i}+1=K_{j}=K_{k}$    **then**     $N_{i}$ adjusts its phase to $\left(1-\alpha \right)\phi_{i}+\alpha \phi_{mid}$    **endIf**    **endIf**  **endFor** **endIf** Each node has occupied a period of the frame       //success to occupy time slots // Conflict detection process **If** the collision occurs between nodes $N_{i}$ and $N_{J}$ in the one-hop    **then**       //punching   $N_{i}$ and $N_{j}$ calculate the punching frame based on $M_{i}$ and $M_{j}$  **In next period**   $N_{i}$ and $N_{j}$ keep listening in the frame they select **else**   the collision occurs between nodes $N_{i}$ and $N_{J}$ in the two-hop       //neighbor detection   $N_{k}$ is the junction node of $N_{i}$ and $N_{j}$   $N_{k}$ send collision-warning packet to $N_{i}$ and $N_{i}$ backs off **endIf** **endFor**


According to the rule, the node *i* prepares to adjust when the phases of its neighbors have been collected. Using these information, node *i* adjusts its phase before it fires again. The equation can be presented as:

(5) $$\begin{array}{cc} \; & \phi_{mid}=\frac{1}{2}\left[\phi_{previous_{i}}+\phi_{following_{i}}\right]\\ \; & \phi_{i}^{\prime }=\left(1-\alpha \right)\phi_{i}+\alpha \phi_{mid} \end{array}$$

where α∈(0,1) is a parameter that balance the step length in one adjustment. $N_{i}$ jumps from $\phi_{i}$ to $\phi_{i}^{\prime }$ and its time stamp is from $\theta_{i}^{k}$ to $\theta_{i}^{k+1}$.

For further explanation, we take Figure 3 as an example, the adjustment process is as follow: initially, all nodes set their Ts(0) to 0. When $\phi_{c}$=1, node *c* reaches the firing point. In the meanwhile, node *b* is in the case 2 and node *d* is in the case 3, so node *b* will jump firstly and its time stamp become to $Ts_{b}\left(t\right)=1$. In the next period, when node *c* fires again, node *b* keeps its time stamp so that node *d* meets the adjustment condition to jump to the midpoint of its neighbors and its time stamp become to $Ts_{d}\left(t\right)=1$. As the time stamps of all nodes are the same, the process will go on. Therefore, the time stamp can limit the negative effects of stale information.

The protocol has several key features:

*(1) Guaranteed convergence to collision-free state*

The system provably converges to a state in which all nodes are spread out with a spacing of T/n at least and exits no collision, regardless of the initial state.

*(2) Solve the stale information*

In the protocol, every node adds a time stamp in its frame, and we set a rule about the order of adjustment, as mentioned above, to prevent convergence performance from degrading.

*(3) Self-adapting*

If the topology changes due to nodes add or remove or multi networks merge, the system does not keep desynchronized, and the network will be chaos eventually. We propose a mechanism named punching algorithm which solves the problem about part of the topology suffering from the collision, and we will describe the punching process in next section.

## 4. Implementation Issues

### 4.1. Stability of the Desynchronization Process

**Figure 4:**
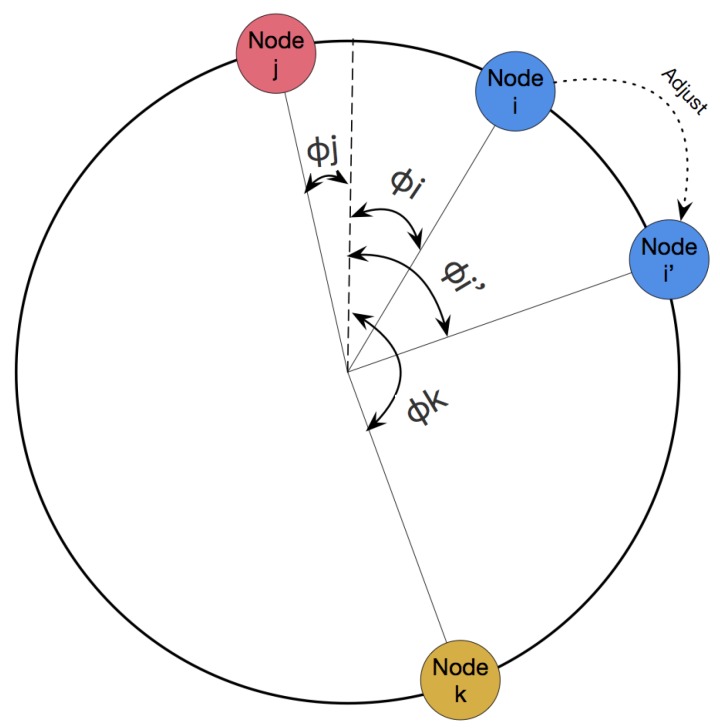
A simple adjustment process.

The aforementioned protocol guarantees that a node will never fire outside its own slot. Note that if this were not the case, node i can be unable to send its firing message since the channel would be occupied by its neighbor. To see that this is the case, we will consider the local behavior for a set of nodes: *i*, *j* and *k* (as seen in Figure 4). We can see that the $\phi_{i}$ in round k+1 is contained by its own time slot in round *K*:

(6) $$\begin{array}{c} \phi_{j}<\phi_{mid}=\frac{1}{2}\left(\phi_{j}+\phi_{k}\right)<\phi_{k} \end{array}$$

(7) $$\begin{array}{c} \phi_{i}^{\prime }=\left(1-\alpha \right)\phi_{i}+\alpha \phi_{mid} \end{array}$$

(8) $$\begin{array}{c} \left(1-\alpha \right)\phi_{i}+\alpha \phi_{j}<\phi_{i}^{\prime }<\left(1-\alpha \right)\phi_{i}+\alpha \phi_{k} \end{array}$$

If α∈(0,1)

(9) $$\begin{array}{c} \phi_{j}<\alpha \phi_{j}<\phi_{i}^{\prime }<\alpha \phi_{k}<\phi_{k} \end{array}$$

Where $\phi_{i}^{\prime }$ means the phase of node i after adjustment. The Equation (9) implies that when $N_{i}$ adjusts, it will always jump to a point between $N_{j}$ and $N_{k}$.

### 4.2. Self-Adaptation of MD-MAC

It is normal in the ad hoc network that a crop of node adds to or removes from the topology, so that the state of the network will be not stability. For the single-hop network, all collisions happen in the range of one hop. But for the distributed network, we usually divide the collision into two categories: one-hop collision and two-hop collision. In this part we focus on the collision detection in the FANET situation where multiple topologies merge after the network converges and propose two schemes to solve these problems respectively.

#### 4.2.1. One-Hop Collision

**Figure 5:**
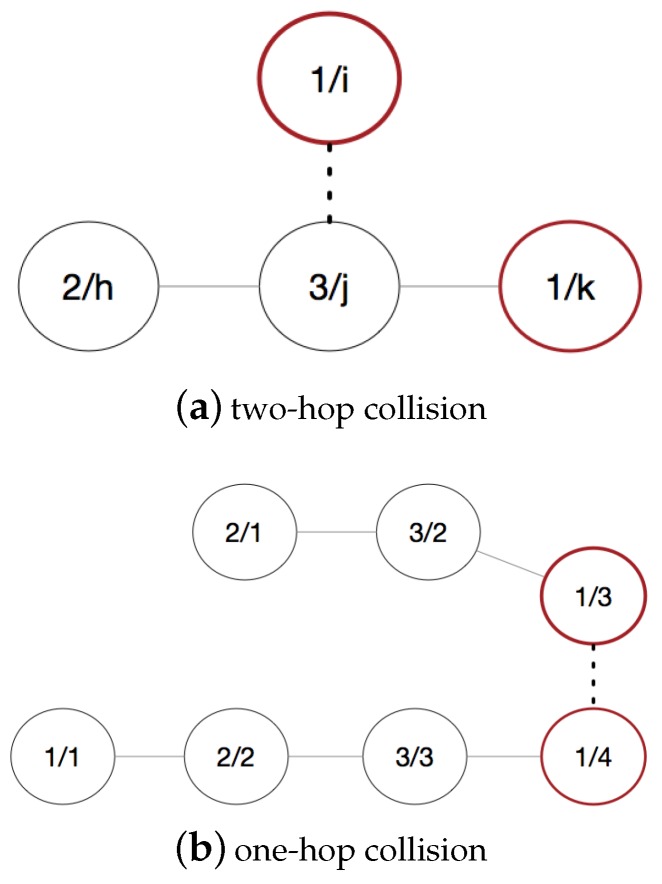
Collision model.

As described in Figure 5b, there are two topologies consisting of four nodes and three nodes separately. The number in the left and right in the cycles means that sequence number and slots occupied by nodes respectively. Assuming that these two topologies close to merge and node 4 and node 3 which utilize the same time slot reach the communication range. Therefore, in the slot 1, collision occurs when two nodes send packets simultaneously.

**Figure 6:**
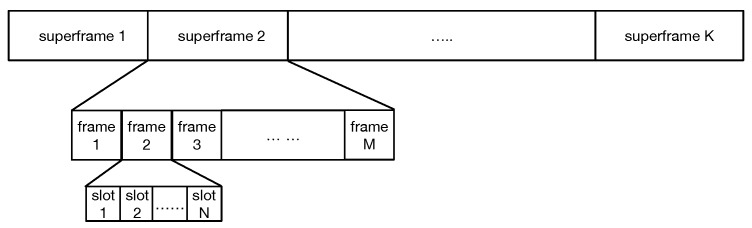
The structure of period.

As shown in Figure 6, the format of the frame consists of *K* superframes including *M* frames, and each frame consists of *N* time slots (according to the topology, $N\leq T/(D_{max}+1)$). It is a simple process that we choose different frame *i* in each superframe for every node. In frame *i*, nodes stop sending packets in their slots and keep listening. For example, in Figure 5b, node 3 and node 4 choose frame *a* and frame *b* as their punching hole frame in the superframe *m*, node 3 will listen to the transmission of node 4 in slot 1, so as node 4. After that, node 3 and node 4 will know the collision and back off to choose slot again.

For balancing the convergence speed and throughput, the punching period is related to degree and time. Known the max degree in the network, nodes utilize sine or cosine model randomly, the model can be expressed as:

(10) $$M_{i}=\left\{\begin{array}{c} \alpha F(sin\left(\eta_{i}\mathrm{Softmax}\left(d_{i}\right)\pi /2\right))\\ \alpha F(cos\left(\eta_{i}\mathrm{Softmax}\left(d_{i}\right)\pi /2\right)) \end{array}\right.$$

(11) $$\alpha_{k+1}=\left(a\alpha_{k}+b\right)\left(modM\right)$$

where α is calculated by LGC (Linear Congruential Generator, finction (10)), and $d_{i}$ is the degree of node *i*. The Softmax is:

(12) $$S_{i}=\frac{e^{i}}{\prod_{j}e^{j}}$$

So each node can get a random punching number with a high uniformity according to Equation (9) corresponded to the sequence number of frame.

#### 4.2.2. Two-Hop Collision

If the collision occurs between nodes in the two-hop communication range, we can use the junction node to detect the collision. As shown in Figure 5a, node *i* joins in the topology and sends packet to node *j*, so that the collision will occur between node *i* and node *k* in slot 1. Before the collision happens, node *j* can receive packet from node *k* in slot 1 every period. So if node *j* cannot receive packet in slot 1, there are two reasons that node *k* removes from the topology or node *j* suffers from collision. Then node *j* will send a packet to node *k* and warns it the collision. Therefore, node *i* occupies slot 1, and node *k* backs off and selects idle slot in the next period.

## 5. Evaluation

**Figure 7:**
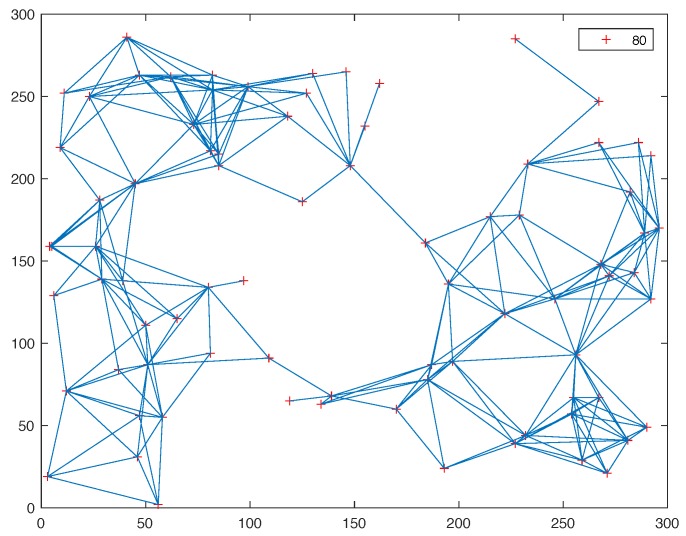
The Topology Structure.

The performance of the proposed MD-Mac protocol is evaluated by extensive simulation experiments compared with the existing distributed TDMA and collision avoidance protocols in different ways, including Reins-Mac [19], Z-MAC [16], improved scheduling policy with RL-model, L-Mac [20], L-BEB [21] and so on. In the multi-hop network, what the most matter is the performance of throughput and convergence speed of the whole network, which is the focus of this evaluation. In addition, for the Ad Hoc network, the energy consumption during the resource allocation is a fraction of the whole. Thus, the energy problem is not the core in the evaluation. The point of collision-free scheme we focus on is the detection speed. Our proposed protocol can apply in the random topology. In the evaluation, we simulate the different topology scenarios where nodes are randomly distributed in a 300 m × 300 m plane. one of the topologies, which concludes 80 nodes and the interference range is 80 m, is shown in Figure 7 and the dotted line represents a connection link between two nodes. The specific simulation parameters are shown in Table 1. The system yields an average target density of 2 to 20 one-hop neighbors through changing the interference range for each node. Without specific description, the results presented in this section are the average values of 1000 Monte-Carlo simulations.

### 5.1. Example of the MD-MAC Operation

**Figure 8:**
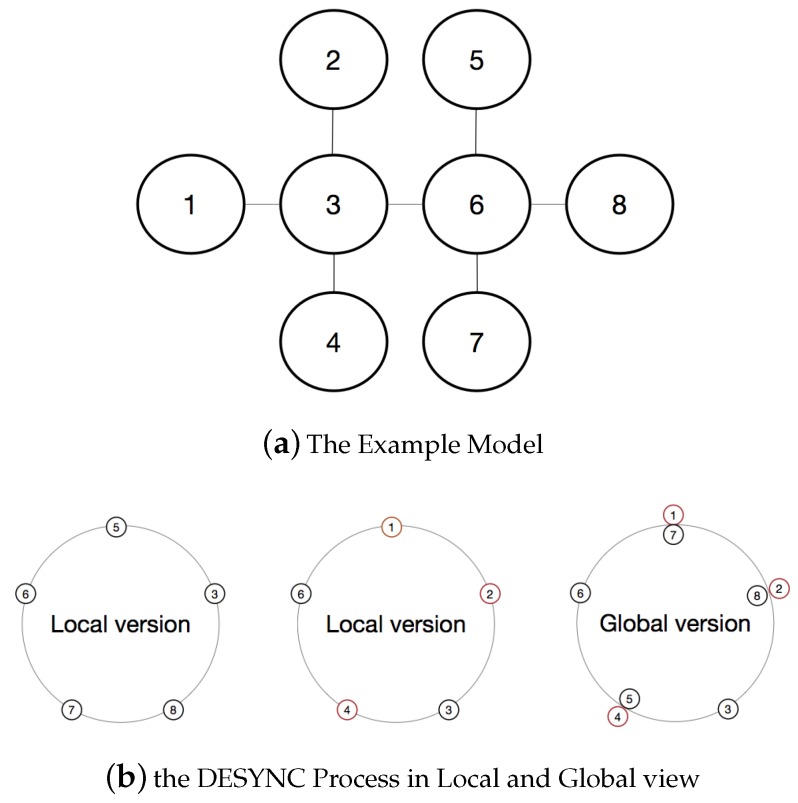
The model of example topology.

We first present an example to show the MD-MAC operation which obtains the stable state. Figure 8a shows the initial topology of 8 nodes. In the local view of node 1, we can see five nodes in the ring (as shown in Figure 8b). After the MD-Mac process, the ideal ring of global view will be shown as Figure 8b. It is obvious that the period can be utilized by all nodes based on the topology structure, which gains an advantage on throughput.

### 5.2. Convergence Speed

**Figure 9:**
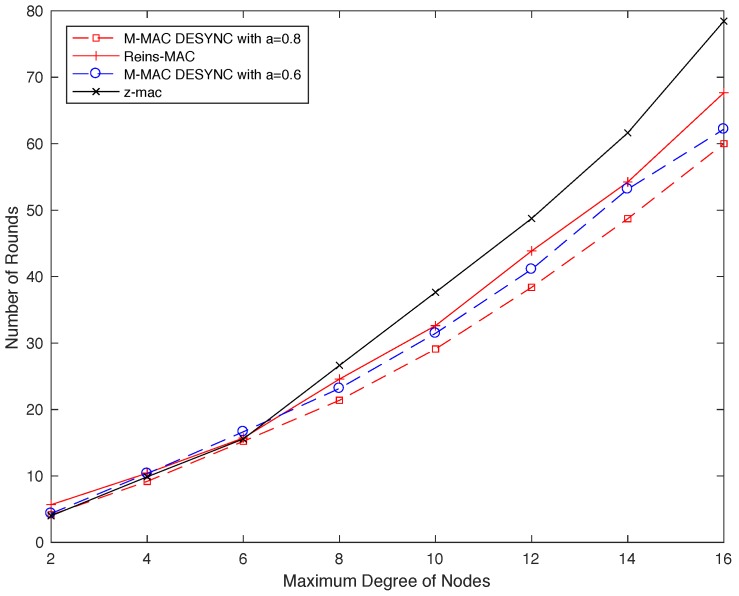
The average number of convergence rounds in different size of maximum degree of nodes in the network.

In order to compare the convergence speed for different protocols, we set different maximum number of nodes in the topology. For the slot allocation process, the convergence time is proportional to the number of the maximum degree of nodes, so their trends are similar. To analyze convergence speed of the MD-MAC, each node sets a schedule to store phases of all nodes and the length of time slots in the simulation. When the length of slots varies less than 5% in the network, it means that the node gets the stable state. Figure 9 plots the simulation results for Reins-Mac, MD-Mac and Z-Mac. All nodes start at the same time without any initial synchronization. As we can see from the picture, networks converge on average less than 80 rounds. As the network density increasing, the speed of Z-Mac is slower and slower, since the randomness of the distribution leads to increased conflict. And the MD-Mac with a=0.6 has the similar convergence speed with Reins-Mac initially, while MD-Mac with a=0.8 is faster than other protocols distinctly. A larger variate a means that in each round all nodes will adjust with a larger step, and the result shows that MD-Mac can contain a fast convergence speed if we set a suitable variate.

**Figure 10:**
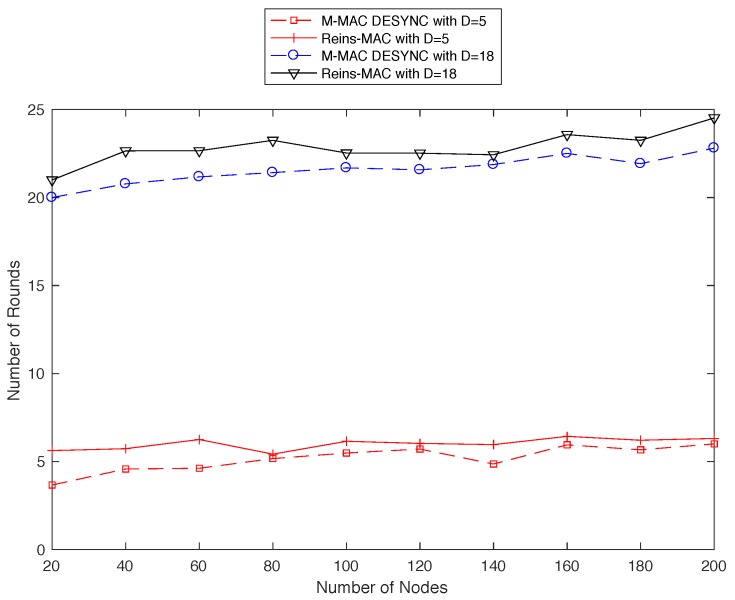
The average number of convergence rounds in different size of network.

We also consider the convergence speed of different number of degree (as shown in Figure 10). The topology we set up will be controlled to keep the number of degree to 5 to 18. It is obvious that as one-hop neighbors increasing, the convergence speed will drop and the number of convergence round floats in a small range. From the above two figures, we can draw a conclusion that the performance of convergence speed of MD-MAC can be increased by more than 15%, and the factor which impacts the convergence speed is the number of nodes. As the topology size increasing, the speed will be low slightly, and the efficiency of MD-Mac is better than Reins-Mac.

### 5.3. Throughput

Another important issue in the distributed network is throughput, which is the ratio of all nodes collision-free transmission time to the transmission cycle T, under saturated scenario with access delay constraints. In particularly, three fixed time slot allocation protocols are compared with our protocol. We set different topology size, where the density of neighbor in the network will be 4 to 10.

**Figure 11:**
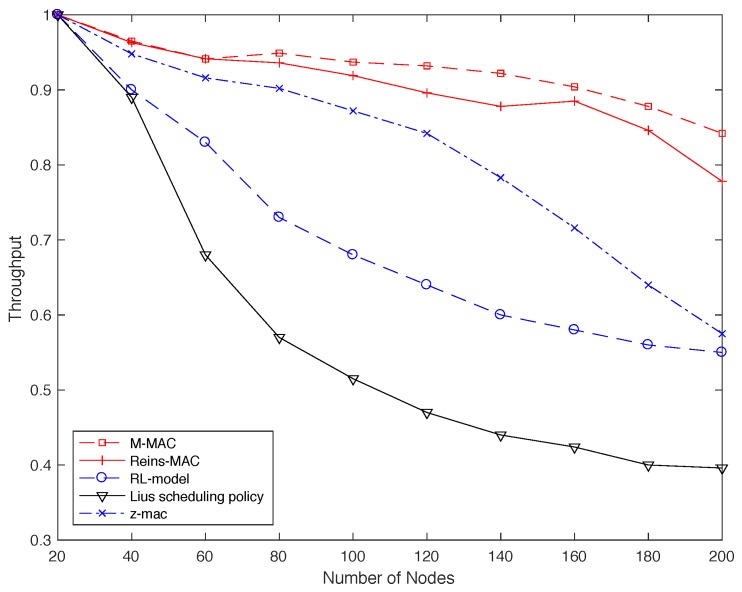
The achieved throughput versus the number of nodes.

Figure 11 shows that the continuous allocation model manifests larger gains than the fixed ones. We can observe that as the increase of the topology size, the throughput of all protocols becomes low. The throughput of RL-model and Lius scheduling policy decreases sharply, since the continuous time slot request causes a sharp increase of the collision in the competition way when the neighbor nodes are too many. With the number of marginal nodes increasing and the collision caused by larger density distributed topology, the throughput of Z-Mac also decreases gradually. At the first three size, the Reins-Mac reaches a similar throughput with our protocol, But the gap between Reins-Mac and MD-Mac becomes larger as the size enlarging. That is because that MD-Mac exploits the continuous overleap slots and all nodes in the protocol connect with their neighbors much tighter due to the time stamp, so that it can get a large throughput, up to 100% more than other protocols.

### 5.4. Convergence Speed of Collision Avoidance

In order to simulate the convergence speed of collision avoidance, we choose ZC, L-BEB, L-ZC, L-MAC and so on as the reference schemes. The range number of contending nodes is set from 2 to 20, and the period length keeps the same for all schemes. The number of frame we set equals to $D_{max}$/2, and the number of length of slots is continuous based on the topology. The result is simulated in the distributed network.

**Figure 12:**
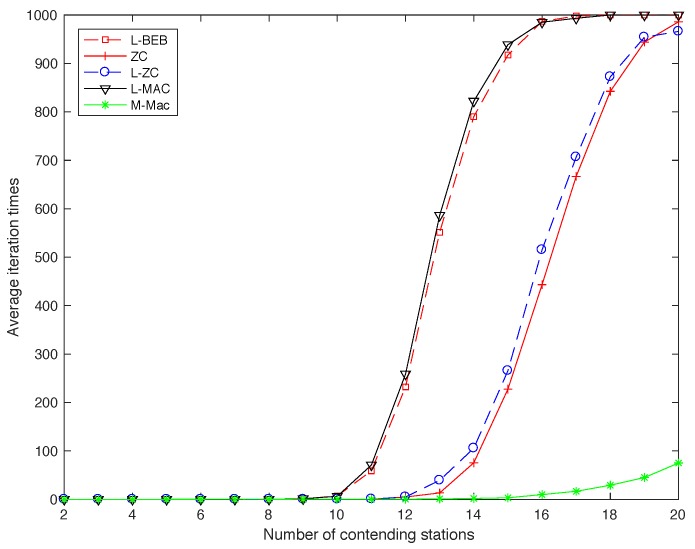
Collision-free speed of MD-MAC.

Figure 12 plots the simulation results for all schemes. We can observe that, in general, the reference schemes (i.e., ZC, L-BEB, L-ZC and L-MAC) have significant drawback of low convergence speed that their convergence time increases sharply with the number of contending nodes. Note that two-hop collisions can be solved in several periods and one-hop collision can be detected after nodes listen to others, so the fast convergence speed of MD-MAC comes partly from the choice of listening frame algorithm. It is obvious that the performance of L-ZC and ZC is better than L-BEB and L-MAC, due to the fact that in ZC and L-ZC, the idle slot has more probability to be selected, and therefore has less probability to collide. It is clearly shown that the detection speed of MD-MAC is much faster than other schemes.

## 6. Conclusions

In this paper, we propose a distributed TDMA protocol based on Desynchronization process, i.e., MD-MAC. The MD-MAC allocates time slots according to the phase of each node. The node judges itself whether adjusting its phase to the midpoint of its neighbors based on the time stamp rule. And the process stops until the whole network keeps stable. it can effectively avoid the adverse impact of stale information. We also propose an effective collision-free scheme including punching process and neighbor detection which solves the collision on account of topology changing or multiple network remerging after leaving. The MD-MAC protocol works in a full distributed manner, requiring no global information or central coordination and needs a low computation power. The simulation shows that MD-MAC protocol has better performance than existing protocols in terms of convergence speed, throughput and collision avoidance. 

## Figures and Tables

**Table 1:** Simulation Parameters.

link capacity	2 Mbps
Broadcast Range	60–120 m
Transmission power	1–2 mW
Range of neighbor nodes	[2, 20]
Number of nodes	[20, 200]
Average transfer delay	0.5 ms
The number of minimum slot	100
